# Retraction: miR-551b regulates epithelial-mesenchymal transition and metastasis of gastric cancer by inhibiting ERBB4 expression

**DOI:** 10.18632/oncotarget.28766

**Published:** 2025-08-29

**Authors:** Guangyuan Song, Hongcheng Zhang, Chenlin Chen, Lijie Gong, Biao Chen, Shaoyun Zhao, Ji Shi, Ji Xu, Zaiyuan Ye

**Affiliations:** ^1^The Second Clinical Medical College, Zhejiang Chinese Medical University, Hangzhou, Zhejiang, China; ^2^The First Clinical Medical College, Zhejiang Chinese Medical University, Hangzhou, Zhejiang, China; ^3^Department of Gastrointestinal and Pancreatic Surgery, Zhejiang Provincial People’s Hospital, Hangzhou, Zhejiang, China; ^4^Key Laboratory of Gastroenterology of Zhejiang Province, Hangzhou, Zhejiang, China; ^*^These authors contributed equally to this work and are co-first authors

**This article has been retracted:** Oncotarget has completed the investigation of this paper. Our Image Forensics analysis revealed that the immunofluorescence images of MNK45 cells in Figure 6A–6D overlapped with Figure 4A–4D images of AGS cells from an unrelated paper [1], previously submitted to a different journal. The authors requested a correction and provided the original, correct images. However, the Oncotarget Scientific Integrity Office was informed of a recent (June 2025) investigation by the National Natural Science Foundation of China (NNSFC) of several papers published in different journals. The NNSFC concluded that this article, “published by Xu Ji, Song Guangyuan and others from two universities in Zhejiang Province contained problems of image forgery and tampering.” Considering these findings, the editorial decision has been made to retract this paper. All authors were informed of this retraction, but we did not receive a response from them.

Original article: Oncotarget. 2017; 8:45725–45735. 45725-45735. https://doi.org/10.18632/oncotarget.17392
